# Early career interview: Viviana Mucci

**DOI:** 10.4155/fsoa-2019-0015

**Published:** 2019-05-03

**Authors:** Viviana Mucci

**Affiliations:** 1 Department of Neurology, University Hospital Zürich, Frauenklinikstrasse 26, 8091, Zürich, CH; 2 Research & Rehabilitation, Swiss Concussion Center, Schulthess Klinik, Lengghalde 2CH-8008, Zürich, CH

**Keywords:** interview, neurotology, researcher

Viviana Mucci was the winner of the Early Career Research Award 2018. Read her interview below to find out about her career.

## Please tell us about your career history to date

I am originally from Italy. In 2008, I moved to London, where I started my journey into physiology. I graduated in 2012 from a Bachelor of Science course in Medical Physiology at the University of East London, UK. Throughout this journey, I discovered my strong passion for extreme physiology; so I was lucky enough to get admitted to one of the few Space Physiology courses in the world, at King’s College London, UK. I completed my Master of Science in Space Physiology & Health in 2013. During that time, I had amazing experiences, such as a placement at the Royal Air Force and at the University of Jean Monnet in France. After this more research-based type of experience, I wanted to expand my clinical knowledge, and I started working at Great Ormond Street Hospital, London, UK in 2014 as a Clinical Physiologist in the Sleep Respiratory unit. Soon after I got the opportunity to move to Belgium and work as a Research Entrepreneur in the field of Cardiology at Erasmé Hospital, in Brussels. I have a strong interest in emerging technology and this was the perfect opportunity to develop that. I was part of a team that created a Spin-Off project with the Université Libre de Bruxelles and the European Space Agency. I was working on cardiac monitoring devices, and my job involved acquiring information from engineers in the R&D department and translating them for investors and physicians testing our technology. We developed a new wearable and portable cardiac monitoring device. Together with my team, we oversaw the clinical trials and developing a successful business strategy. During this experience, I learnt how to communicate effectively with a diverse range of people from venture capitalists to healthcare professionals. This experience taught me how to deal with the many challenges of a Spin-Off and how to manage people from different backgrounds.

In 2015, I received an invitation to pursue a PhD program at Antwerp University, Belgium. I had always wanted to do a PhD and to work in the research field. My PhD focused on two vestibular disorders named: Mal de Debarquement Syndrome (MdDS) and Visually Induced Dizziness. I was able to gather epidemiological information about MdDS, given that it is currently considered a new and rare disorder. Together with other senior researcher in this field we tried to designed and improved the diagnostic criteria for MdDS; and further validated a potential treatment, based on optokinetic stimulation. Given the high number of female subjects suffering from both conditions, a clinical trial specifically designed around hormonal imbalances was also implemented. For these projects, I also established an international collaboration with Western Sydney University, Australia and Ichan School of Medicine, Mount Sinai, New York, USA. Their support was essential to bring these projects to success. Especially with Dr. Cherylea Browne, my promoter from Western Sydney University, who was a great mentor and provided me with valuable knowledge and expertise in the field.

As part of my PhD, I mentored several master and bachelor students and delivered lectures in different universities across Europe. I also worked in a European funded project named EMBalance, which united expertise from seven different European countries to address the challenging problems related to imbalance disorders. We developed a decision support system for primary and secondary care physicians, for diagnostic evaluation, behavior prediction and effective management planning of balance problems. We wanted to reduce the number of misdiagnoses and to improve the management of such diseases. My passion for healthcare has also led me to the European Health Parliament. In 2015, I became the Chair of the committee ‘Migration and Health Challenges’. Together with my colleagues, we worked on a new policy in order to address some of the main issues that the European healthcare system faces currently.

Currently, I am working as a postdoc at the University Hospital Zurich and I work for 50% of my time at the Swiss Concussion Centre (Zurich, CH). While also providing consultancy for MdDS treatment at Geneva University Hospital, (in Geneva, CH). Thus, my job is divided between clinical work and research. For the research part I am continuing working on Mal de Debarquement Syndrome and Visually Induced Dizziness (VID) and I am also working in partnership with University of Newcastle, Callaghan University in Australia about the use of cannabinoid-based drugs (CBD) on reducing Motion Sickness. For the clinical part, I am trying to create novel rehabilitation programs for patients with vestibular insults and post concussion syndrome, mostly using optokinetic stimuli.

## What made you choose a career in your field?

I became passionate about the vestibular system (balance system) during my Master of Science in Space Physiology & Health. The balance system is probably one of the most sophisticated systems we have. When cosmonauts or astronauts are exposed to microgravity the vestibular system is greatly disrupted. As soon as they enter space, astronauts and cosmonauts are subjected to the so-called space motion adaptation, which is a transient period where they are exposed to disorientation, nausea, vomiting and sensory conflicts. I studied how this ancient system could be so important and relevant for our well-being in space as on Earth. Following space research, where I was assessing cosmonaut vestibular functions pre- and post-flight, I had the opportunity to work closely with patients. When I started working in the clinical field of the vestibular system I really understood that this is what I wanted to do in life. These patients suffer not only from a debilitating condition, but they often struggle to get recognition as well as diagnosis and management. Although our vestibular knowledge has grown over the last few decades there are still many pieces of this remarkable system that remain to be addressed. This lack of knowledge is a strong drive for me to continue pursuing research in this particular field.

## Describe the most difficult challenge you have faced & how you overcame it?

The biggest challenge I faced was completing my PhD. As most PhD students find, a PhD can be overwhelming at times. I was lucky to get amazing support and great guidance from my promoters, which helped me in the darkest times. The challenges I faced and overcame have served to enhance my passion for research and emphasise the importance of collaborative work, especially when researching less common conditions.

## How do you feel you have impacted your field?

Via an international collaboration I was able to gain the largest dataset ever collected on MdDS subjects using a series of retrospective questionnaires. From these results we created new diagnostic guidelines and were able to have more data about MdDS-associated co-morbidities. For the first time, my research established a link between the great female preponderance and MdDS. We were able to show an interrelation between hormonal fluctuations and symptom changes in female MdDS patients. These results are novel and have not been demonstrated before. MdDS patients reported symptom aggravation during menses and mid-cycle. Thus, we described a potential hypothesis, which requires further evaluation. We hypothesised that the estrogen withdrawal, occurring during menses, may trigger an increase in MdDS symptoms. We also gained valuable information about female MdDS patients, for example, that onset age should be further evaluated as a risk factor, matching with menopausal and perimenopausal phases. In addition to this, for the first time, we also collected data from MdDS women who had been pregnant or were currently pregnant while having MdDS symptoms. The study was performed by means of a retrospective questionnaire. During the 9 months of pregnancy, the majority of our respondents reported an improvement in symptoms, which could further confirm the involvement of hormonal changes in MdDS symptomatology and pathophysiology. As part of my research, I also focused on one of the current treatment for MdDS. Together with my team we examined if there was a placebo effect with the optokinetic treatment (OKN), proposed by the team (Dr Dai, Dr Yakushin, Dr. Cohen) from Ichan School of Medicine, Mount Sinai, NY, USA. From our data, no placebo was recorded, while we were able to obtain similar results to the team from New York. Thus the optokinetic treatment was effective in reducing MdDS symptoms also in our study. However, we discovered that some symptoms remain after the treatment such as visually induced dizziness and migraine in some patients. Thus this has led us to continue to research MdDS further. I think that my work has been important mainly because it could help patients’ access optokinetic treatment in Europe and because it has highlighted aspects of MdDS that require more research.

## What are your aims for the future?

I am aiming to continue in the science field, despite the struggle with available fundings for supporting research. I hope to continue my work on MdDS as well as to further advance the knowledge of vestibular dysfunctions in concussed patients.

Particularly with regards to MdDS, I am hoping to continuing researching a potential hormonal link within MdDS pathophysiology and further assessing MdDS treatments. I am also hoping to assess cognitive impairment in MdDS patients with cognitive tests assessment before and after a successful treatment. I am determined to understand the link between visual sensitivity/visually induced dizziness and binocular vision dysfunction and MdDS.

More generally, I want to continue researching MdDS and vestibular patients, to bring innovative treatments options that can reduce patients’ symptoms and to improve patient care.

## What advice do you have for those hoping to win the award in the future?

There is no one way to write a good application to win this award. My best advice is to make your application unique and to use a distinctive style. Provide a unique insight into your research and experiences as well as your future vision and aspirations. This uniqueness is the key and will help voters to choose you. Show your personality, delve deep into your passions for your research and demonstrate how they have shaped your work and will shape your future. Showing a clear response that can only ever relate to you and your work will help voters and the jury to vote for you.

